# Does central executive training and/or inhibitory control training improve emotion regulation for children with attention-deficit/hyperactivity disorder? A randomized controlled trial

**DOI:** 10.3389/fpsyt.2022.1034722

**Published:** 2022-12-06

**Authors:** Nicole B. Groves, Elizabeth S. M. Chan, Carolyn L. Marsh, Fatou Gaye, Emma M. Jaisle, Michael J. Kofler

**Affiliations:** ^1^Department of Psychology, Florida State University, Tallahassee, FL, United States; ^2^Department of Psychology, Florida International University, Miami, FL, United States

**Keywords:** attention-deficit/hyperactivity disorder (ADHD), emotion regulation, working memory, inhibitory control, executive function

## Abstract

**Introduction:**

Approximately 48–54% of children with attention-deficit/hyperactivity disorder (ADHD) have impairing difficulties with emotion regulation, and these difficulties are not ameliorated by first-line ADHD treatments. Working memory and inhibitory control represent promising intervention targets given their functional, if not causal, links with ADHD-related emotion dysregulation.

**Methods:**

This preregistered randomized controlled trial tested whether two digital therapeutic training protocols that have been previously shown to improve working memory (Central Executive Training [CET]) and inhibitory control (Inhibitory Control Training [ICT]) can improve emotion regulation in a sample of 94 children with ADHD aged 8–13 years (*M* = 10.22, *SD* = 1.43; 76% White/non-Hispanic; 29 girls).

**Results:**

Results of Bayesian mixed model ANOVAs indicated both treatment groups demonstrated significant decreases in emotion dysregulation relative to pre-treatment at immediate post-treatment (parent report; *d* = 1.25, BF_10_ = 8.04 × 10^13^, *p* < 0.001), at 1–2 months after completing treatment (teacher report; *d* = 0.99, BF_10_ = 1.22 × 10^6^, *p* < 0.001), and at 2–4-months follow-up (parent report; *d* = 1.22, BF_10_ = 1.15 × 10^14^, *p* < 0.001). Contrary to our hypotheses, the CET and ICT groups demonstrated equivalent reductions in emotion dysregulation and maintenance of effects. Exploratory analyses revealed that results were robust to control for informant expectancies, ADHD medication status/changes, in-person vs. at-home treatment, child age, and time from treatment completion to post-treatment ratings.

**Discussion:**

To determine whether working memory and inhibitory control are causally linked with ADHD-related emotion dysregulation, future studies should include active control conditions that do not train executive functions prior to making decisions about the clinical utility of CET/ICT for the treatment of emotion dysregulation in ADHD.

**Clinical trial registration:**

[https://clinicaltrials.gov/], identifier [NCT03324464].

## Introduction

Attention-deficit/hyperactivity disorder (ADHD) is a neurodevelopmental disorder characterized by impairing symptoms of inattention, hyperactivity, and impulsivity (1). ADHD affects approximately 5% of school-aged children (2), and most children with ADHD experience difficulties with emotion regulation that, in turn, portend significantly greater distress and impairment than ADHD symptoms alone (3). Unfortunately, the limited available literature suggests that evidence-based treatments for ADHD (4)—including psychostimulants (5) and behavioral parent training (6)—often do not reduce emotion dysregulation, suggesting the need for interventions that directly target factors that underlie ADHD-related emotion dysregulation. Working memory and potentially inhibitory control have been linked functionally with ADHD-related emotion dysregulation (7–10), suggesting that they may reflect promising intervention targets for producing downstream improvements in emotion regulation for children with ADHD. The present randomized controlled trial tests the extent to which two cognitive training protocols that have been previously shown to improve working memory (Central Executive Training [CET]) and inhibitory control (Inhibitory Control Training [ICT]) (11) can improve emotion regulation for children with ADHD.

### Emotion regulation and executive functioning in attention-deficit/hyperactivity disorder

Emotion regulation refers to the ability to modulate the speed and intensity of emotional escalation and de-escalation, and it involves complex physiological, experiential, and behavioral processes (12, 13). Approximately 48–54% of children with ADHD exhibit comorbid difficulties with emotion regulation based on recent meta-analytic evidence [*d* = 0.80–0.95; (3)]. Mitigating emotion dysregulation in ADHD is imperative given that it increases the already large burden of illness associated with ADHD (14), including predicting greater academic and social impairment (15–17), higher rates of healthcare utilization (16), and higher daily parenting stress (18) than ADHD symptoms alone (19). Additionally, emotion dysregulation persists into adulthood for many people with ADHD (20–22), and portends increased risk for the development of comorbid psychopathology [e.g., oppositional defiant disorder, anxiety, depression; (23)].

Several conceptual models have been proposed to explain the high prevalence and adverse outcomes of emotion dysregulation in children with ADHD (3, 24), with growing acknowledgment that the phenomenology, etiology, course, and correlates of emotion dysregulation are likely as heterogenous as most other ADHD-related symptoms (25–28). Of the proposed mechanisms linking ADHD and emotion regulation, underdeveloped executive functions—particularly working memory and inhibitory control—reflect promising investigative targets. Working memory refers to the active, top-down manipulation of information held in short-term memory (29), and is impaired in 68–85% of children with ADHD (30–32). Inhibitory control refers to a set of interrelated cognitive processes that underlie the ability to withhold (action restraint) or stop (action cancelation) an ongoing behavioral response (33), and is impaired in 21–46% of children with ADHD (28, 34, 35).

Working memory and inhibitory control have each been theorized as core deficits underlying ADHD-related behavioral symptoms and functional outcomes [e.g., (36, 37)], including emotion dysregulation (38). Conceptually, intact executive functions are necessary to regulate the generation and expression of emotion at each of its theorized stages. That is, emotion generation and expression require one to select and modify situations, actively attend to stimuli, form cognitive interpretations of events, and modulate responses accordingly (39). Deficits in working memory and/or inhibitory control may lead to a breakdown in any of these complex processes. Indeed, working memory predicts emotion regulation in samples of children with ADHD with and without common comorbidities (7, 9). Additionally, experimental evidence indicates that increasing working memory demands produces disproportionate increases in negative emotional expression for children with ADHD relative to their typically developing counterparts, suggesting that working memory is functionally related to emotion dysregulation in ADHD (10). Inhibitory control has also been associated with emotion regulation in children with ADHD (9, 40), and there is some evidence suggesting this relation is causal in other populations [i.e., emotionally dysregulated adults; (41)]. Interestingly, however, when considered together, only working memory uniquely predicted emotion regulation, whereas inhibitory control did not (8).

### Executive function interventions for emotion dysregulation in other populations

Given the established links among working memory, inhibitory control, and emotion regulation, there has been increased interest in the extent to which training these executive functions can improve emotion regulation in various populations. For example, training working memory using emotional/affective stimuli has improved emotion regulation in samples of neurotypical adults (42–44) as well as clinical samples of adolescents and adults (45, 46). In contrast, findings are more mixed for inhibitory control, such that training inhibitory control improved emotion regulation in adults with elevated emotional reactivity (41) but did not affect emotion regulation in neurotypical adults (47). Finally, one study trained both working memory and inhibitory control, and reported improved emotion regulation in typically developing preschoolers relative to waitlist controls (48). Taken together, the evidence supporting training working memory and/or inhibitory control for improving emotion regulation in non-ADHD samples is promising, albeit mixed.

### Executive function interventions for emotion dysregulation in children with attention-deficit/hyperactivity disorder

Both working memory and potentially inhibitory control may be functionally related to emotion regulation difficulties in children with ADHD (9, 10), but, to our knowledge, no study to date has examined whether emotion regulation improves in children with ADHD following targeted training of executive functioning (49). In a partial exception, Tamm et al. (50–52) reported that a play-based (non-computerized) metacognitive attention training for preschoolers with ADHD failed to improve emotion regulation based on parent report. Similarly, in a randomized controlled trial comparing the efficacy of training all, some, or no executive functions, impacts on most cognitive and behavioral outcomes were non-specific and not attributable to any particular treatment target (53). Additionally, meta-analytic evidence suggests that, even though direct training may improve working memory and inhibitory control for preschoolers with ADHD and related externalizing behaviors, effects on behavioral outcomes are not significant (54). However, the extent to which similar findings would be obtained by training specific executive functions in school-aged samples of children with ADHD remains unknown.

For children with ADHD, evaluating the efficacy of executive function training protocols is further complicated by target misspecification and related issues of first-generation protocols that significantly limit their potential to produce downstream effects on behavior and functioning [for reviews see (49, 55)]. Specifically, most executive function training protocols have historically not produced the intended improvements in the executive function(s) they were intended to train (49, 56, 57). Indeed, most executive function training protocols have been shown to train cognitive abilities—such as short-term memory capacity rather than central executive working memory processes (29)—that are not impaired in most children with ADHD and, in most cases, are unrelated to ADHD symptoms and functional outcomes even cross-sectionally (11, 49). Thus, their lack of downstream effects on behavior and functioning is unsurprising to the extent that they are training cognitive abilities that generally do not support the behaviors we are trying to modify [e.g., (55)].

To address these limitations, our group created two translational, evidence-based, digital therapeutic treatments that include gaming elements (11, 34, 58). These computerized treatments incrementally increase demands on their target processes (central executive working memory for CET, inhibitory control for ICT). In previous clinical trials, CET and ICT were both rated as acceptable and feasible by parents and children (11, 34). In terms of affecting their respective cognitive training targets, CET was superior to gold-standard behavioral parent training (34) and ICT (11) for improving working memory performance. Similarly, ICT was superior to CET for improving inhibitory control, albeit only on one of two outcome tests, demonstrating that both CET and ICT successfully improve their targeted executive function (11). In terms of clinical outcomes, CET produced improvements in parent-rated ADHD symptoms that were equivalent to those obtained from gold-standard behavioral parent training, and CET was superior to behavioral parent training for decreasing objectively assessed hyperactivity in a sequential recruitment controlled trial (34). In a double-blind randomized controlled trial comparing CET and ICT, CET was superior to ICT in reducing parent- and teacher-reported ADHD symptoms and objectively assessed hyperactivity; CET-related ADHD symptom improvement was also maintained at 2–4 months follow up (11).

### Current study

Given that CET and ICT (a) effectively improve working memory and inhibitory control processes, respectively, that are linked with ADHD-related emotion dysregulation; and (b) have been shown previously to produce clinically meaningful improvements in other behavioral and functional domains for children with ADHD (11, 26, 59), we hypothesized that both CET and ICT would produce improvements in emotion regulation. In addition, given evidence that working memory is better than inhibitory control at predicting emotion regulation for children with ADHD (8), as well as prior evidence that CET produced superior improvements relative to ICT on other behavioral and functional outcomes (11, 59), we hypothesized that CET would be superior to ICT for reducing emotion dysregulation.

## Methods

### Study timeline, randomization, allocation concealment, and masking

**FIGURE 1:**
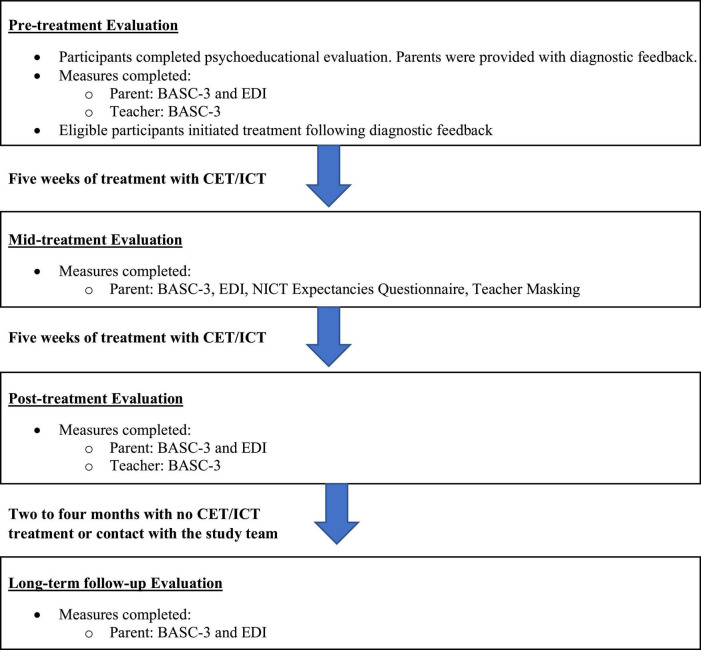
Study timeline. BASC-3, behavior assessment scale for children; CET, central executive training; EDI, emotion dysregulation inventory; ICT, inhibitory control training.

The current study reports on secondary outcomes from a randomized clinical trial of CET vs. ICT for ADHD (11) (Table 1). The sample reflects consecutive referrals from March 2017 to April 2021. Prior to March 2020, children (*n* = 73; 77.7%) completed three visits during the pre-treatment evaluation, lasting approximately 3.5 h each. These children also completed testing sessions at mid-treatment, post-treatment, and 2–4 months follow up. Four treatment cases were lost to follow-up from March to June 2020 as the study was shut down due to COVID-19. Procedures were adjusted when the study resumed in June 2020 to minimize face-to-face contact, including reducing the pre-treatment battery to a single 4-h testing session that included the use of face masks and social distancing for participants and study team members (*n* = 21; 22.3%). Additionally, weekly treatment sessions that occurred in the clinic prior to the shutdown were conducted via telehealth for all children beginning in June 2020, and in-clinic mid/post/follow-up child testing was discontinued. Treatment delivery format (pre-COVID face-to-face vs. peri-COVID telehealth) was probed as a covariate as described below. Parents (pre/mid/post/2–4 months follow-up) and teachers (pre/post) completed measures at each time point according to the original protocol. Teacher questionnaires were sent during the post-treatment session and were completed by teachers approximately 1–2 months post-treatment (Figure 1).

**TABLE 1:** Sample demographic and pre-treatment characteristics.

Variable	ICT		CET		Cohen’s *d*	BF_01_	*p*
	*M*	*SD*	*M*	*SD*			
*N* (boys/girls)	50 (34/16)		44 (31/13)		–	4.15	0.80, *ns*
Age	10.44	1.53	9.96	1.29	0.34	1.43	0.11, *ns*
SES	48.88	9.82	47.06	11.70	0.17	3.42	0.42, *ns*
WISC-V VCI	105.9	1.52	100.82	13.60	0.36	0.86	0.053, *ns*
Race/Ethnicity (W, B, H, MR)	36, 6, 4, 4		35, 5, 3, 1		–	34.48	0.64, *ns*
Medication (no/yes)	34/16		28/16		–	3.77	0.66, *ns*
ADHD presentation (I, H/I, C)	16, 1, 33		14, 1, 29		–	66.67	0.99, *ns*
Comorbidity (no/yes)	18/32		16/28		–	31.25	0.29, *ns*
BASC-3 emotional self-control (total raw score)							
Parent	11.86	6.44	11.34	5.40	0.09	4.26	0.68, *ns*
Teacher	9.44	5.46	11.15	7.40	0.27	2.22	0.21, *ns*
BASC-3 negative emotionality (total raw score)							
Parent	8.60	4.54	8.66	4.03	0.01	4.61	0.95, *ns*
Teacher	4.96	3.46	5.98	4.85	0.25	2.48	0.25, *ns*

B, black; BASC-3, behavior assessment scale for children; C, combined presentation; CET, central executive training; H, Hispanic/Latino; H/I, predominantly hyperactive/impulsive presentation; I, predominantly inattentive presentation; ICT, inhibitory control training; MR, multiracial; SES, Hollingshead SES total score; W, white/non-Hispanic; WISC-V VCI, WISC-V verbal comprehension index standard score; BF, Bayes factor, BF_01_ is the odds ratio of the evidence favoring the null to the evidence favoring the alternative hypothesis. A value of 1 indicates that the data are equally likely under the null and alternative hypotheses, values >1 favor the null hypothesis that the groups are equivalent, and values >3 are considered statistically significant evidence of equivalence.

Randomization was conducted by the study methodologist using unpredictable allocation stratified by medication status according to CONSORT guidelines. Study evaluators were masked to treatment group. Data screening, cleaning, and analyses were conducted masked to treatment group. Best practice guidelines for cognitive training studies were closely followed as described in Table 2.

**TABLE 2:** Critical evaluation of the current study relative to best practice guidelines for cognitive training methodology and reporting standards [adapted from (66) and (75)].

	Criterion/*commentary*
Best practice recommendations from Simons et al. (66)	
√	Assess pre-treatment baseline performance for all groups
	*The current study used a pre/mid/post (parent) and pre/post (teacher) design in which outcomes were assessed at a priori specified time points that included pre-treatment. Pre-treatment performance was assessed and controlled when probing between-group differences at post-treatment.*
√	Include an active, credible control group matched for expectancies
	*Working memory and inhibitory control are both putative core mechanisms implicated in ADHD-related emotion dysregulation. The two versions of the intervention are identical in all aspects except the target mechanism, and served as active, credible controls for each other. The CET and ICT interventions have been shown previously to be identical or not differ significantly in terms of expectancies as well as caregiver- and child-reported feasibility and acceptability* (11).
√	Include at least 20 participants in each treatment arm
	*All analyses include ICT n* = *50 and CET n* = *44 participants.*
√	Randomly assign children to condition
	*Children were randomly assigned using unpredictable allocation concealment.*
√	Pre-register the trial, and explicitly acknowledge departures from pre-registered plan
	*The trial was preregistered [https://osf.io/abwms]. All collected emotion regulation outcomes measures were reported. Data analyses were conducted masked to treatment allocation.*
√	Mask raters for all subjective outcome measures
	*Teachers were masked to treatment status and allocation condition. Caregivers were masked to allocation condition. However, caregivers were not masked to the fact that their child was receiving an intervention because they are active participants in both treatments* (66). *Meta-analytic evidence indicates that estimates of treatment effects are inflated for unmasked raters vs. masked raters by d* = *0.36–0.40 for neurocognitive training studies* (49).
√	Label any analyses conducted after inspecting the data as “exploratory”
	*The analyses reported herein did not depart from the a priori plan, with one clearly marked exception related to an administrative error during data collection for our secondary outcome measure (EDI). Analytic decisions regarding this measure were made based on missing data counts without knowledge of their effects on study results.*
√	Avoid subgroup analyses unless preregistered
	*No subgroup analyses were preregistered; therefore, none were conducted. Within-group analyses were limited to planned comparisons to characterize the pattern of change for each group across assessment points.*
√	Identify all outcome data collected, including outcomes not reported herein
	*A complete list of data collected for secondary research questions can be found on the study’s OSF preregistration website.*
Additional recommendations from Redick (75)	
√	Report full pre-test and post-test means and SDs for all groups
	*Pre-treatment and post-treatment means and* SDs *are shown in Tables 1, 3, respectively.*
√	Provide full, subject-level data as supplementary material
	*JASP (.jasp) data files are posted for peer review on the study’s OSF website [https://osf.io/86fwu/].*
√	Use likelihood ratios, in particular Bayes Factors
	*Traditional* p*-values are supplemented with Bayes Factors to allow stronger conclusions regarding both between-group equivalence and emerging between-group differences.*
√	Examine outcomes graphically to ensure that the pattern of pre- to post-test change is theoretically consistent with the expected pattern of results
	*Graphical representations of study outcomes are shown in Figure 3.*

### Participants

As shown in Table 1, the treated sample comprised 94 children with ADHD aged 8–13 years (*M* = 10.22, *SD* = 1.43; 29 girls) from the Southeastern US, consecutively referred to a university-based research clinic through community resources. Psychoeducational evaluations were provided to caregivers. IRB approval was obtained/maintained; all parents/children gave informed consent/assent. Child race/ethnicity was mixed, with 71 (75.5%) White/Non-Hispanic, 11 (11.7%) Black/African American, 7 (7.4%) Hispanic/Latino, and 5 (5.3%) multi-racial/ethnic children. All participants spoke English.

### Inclusion/exclusion criteria

All families completed a comprehensive evaluation that included detailed semi-structured clinical interviewing [K-SADS; (60)] and age/sex norm-referenced parent and teacher ADHD ratings [ADHD-Rating Scale-5 and Behavior Assessment Scale for Children-3; (61, 62)]. Study eligibility required: (1) DSM-5 diagnosis of ADHD (any presentation) by the directing clinical psychologist and multidisciplinary treatment team based on K-SADS (2013 update for DSM-5) and differential diagnosis considering all available clinical information indicating onset, course, duration, and severity of ADHD symptoms consistent with the ADHD neurodevelopmental syndrome; (2) clinical/borderline elevations on at least one parent and one teacher ADHD rating scale (i.e., >90th percentile), or previous psychoeducational evaluation documenting cross-informant symptoms (e.g., for children prescribed medication that reduces ADHD symptoms at school); and (3) current impairment per K-SADS. Diagnoses comorbid with ADHD in the current sample included anxiety (31.9%), specific learning (22.3%), autism spectrum (13.8%), oppositional defiant (6.4%), and depressive (2.1%) disorders. Additional details regarding the psychoeducational evaluation and differential diagnosis process can be found on our preregistration website: https://osf.io/abwms.

**FIGURE 2:**
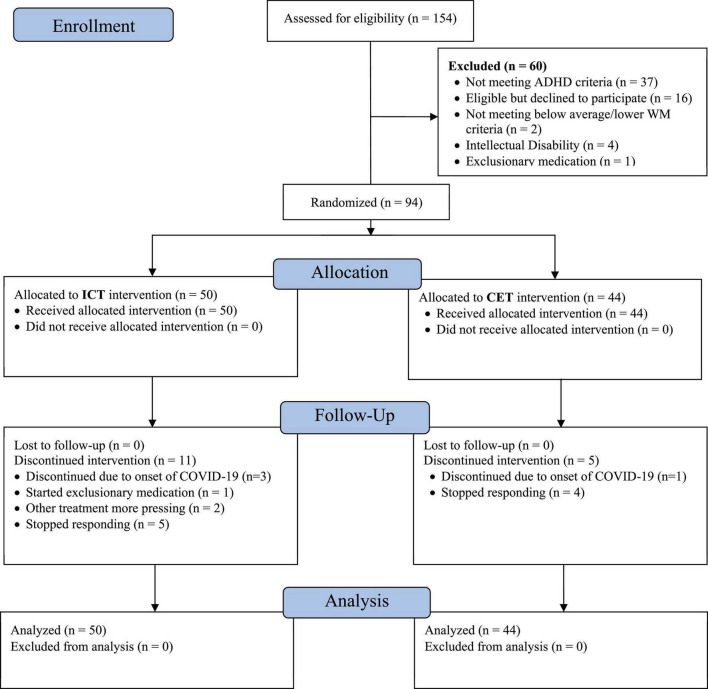
CONSORT diagram. The 154 children assessed for eligibility include all children recruited for evaluation in our research clinic during the study timespan, regardless of recruitment reason (because families would have been offered the intervention trial if their child was diagnosed with ADHD and otherwise eligible). The number of confirmed ADHD cases who were considered for eligibility is 112, of whom 94 (83.9%) were randomized and treated.

As shown in the CONSORT diagram (Figure 2), a total of 112 children with ADHD were evaluated; *n* = 16 were eligible but declined participation, and *n* = 2 were excluded due to average or better performance on all pretreatment working memory tests, resulting in a total treated sample of 94 (83.9% of eligible cases). No inhibitory control thresholds were set as specified in our NIMH grant. Children with ADHD that did vs. did not participate in the treatment phase of the study did not differ on age, sex, SES, race/ethnicity, IQ, medication status, ADHD presentation, and the presence of common comorbidities (all *p* ≥ 0.20). Untreated children with ADHD did not differ from treated children with ADHD on parent-reported emotion dysregulation (*p* = 0.21–0.27), but they had moderately higher teacher-reported emotion dysregulation (*p* = 0.02–0.03; *d* = 0.57–0.61) than the children with ADHD who participated in the treatment phase of the study. Children that were not randomized to CET or ICT were not followed past the pre-treatment evaluation.

Children were excluded from the larger study for gross neurological, sensory, or motor impairment; seizure disorder, psychosis, or intellectual disability; or non-stimulant medications that could not be withheld for testing.

### Procedures

As detailed in Kofler et al. (11), identical procedures were used for both treatment groups. Both CET and ICT are 10-week digital therapeutic treatments accessed via computer or mobile device. Once a week, children were monitored by study staff for a 1-h session while they completed their training exercises in-office (pre-COVID) or via telehealth according to identical, manualized procedures. Additional weekly training sessions were parent-supervised, in-home training (goal: 15-min/day, 2–3 days/week). Weekly in-office (pre-COVID) or telehealth parent check-ins were also included to promote adherence and troubleshoot difficulties with the at-home training. No active treatment components are included in the parent check-ins, which were identical across groups.

### Treatments

Central executive training and ICT each contain nine games, with each game created to train various functions/modalities of their respective targeted executive function. Both treatments include an automated token economy in which children receive virtual “tickets” for successful responses during games, completing games, and completing the daily “mission mode” to facilitate increased engagement in the task and reinforcement of training targets. Tickets were exchanged for tangible prizes that children collected during weekly in-office sessions or intermittently throughout treatment if they participated via telehealth due to COVID-19. The daily “mission mode” consists of three games that the child has not recently played that are selected by the software and must be completed prior to having access to all nine games, and it is designed to ensure appropriate breadth of training. Please see Kofler et al. (34) and Kofler et al. (11) for a more detailed description and rationale of the treatments’ active control, adaptive training, and methods for maximizing dosage. Both interventions have been shown to have high feasibility and acceptability in terms of high parent satisfaction, high child-reported ease of use, and total child training time (11, 34).

#### Central executive training

The computerized CET protocol focused on improving children’s working memory (11, 34). CET contains nine games that train each of the three primary central executive processes–updating, dual-processing, and temporal/serial reordering (30)–using three different stimulus modalities—verbal/phonological, visual, and spatial. CET’s algorithms facilitate continually adaptive training by dynamically adjusting various parameters depending on the training target to incrementally increase central executive demands. Such parameters include target density, categories: stimuli ratio, target: non-target stimuli ratio, visual discriminability, and search space size. For example, increasing the search space size produces greater visual saccades, which, in turn, increases central executive demands during spatial working memory tasks because these saccades interrupt spatial rehearsal (63, 64).

#### Inhibitory control training

The computerized ICT protocol focused on improving the “action restraint” and “action cancelation” components of inhibitory control (65). ICT was developed as an active, credible control comparison for CET based on best practice guidelines for rigorous digital therapeutic treatment trials (66). As such, each of the 9 ICT games contains an identical website address, name, art, animations, storyline, layout, interface, and use of adaptive training algorithms as its CET counterpart. Similarly, ICT dynamically adjusts parameters such as go:stop target ratio, presentation rate, response speed (timers), and number of stimuli (65) to ensure incremental increases in inhibitory control demands. For example, stretching the target density (i.e., increasing the proportion of “go” trials) increases inhibition demands by increasing prepotency, which makes it more difficult to inhibit during infrequently occurring “stop” trials (67).

### Secondary intervention outcome assessment (emotion regulation)

#### Behavior assessment scale for children-3

The BASC-3 (62) contains two subscales that assess emotion regulation based on parent and teacher report: *Emotional Self-Control* and *Negative Emotionality*. The emotional self-control subscale assesses children’s skill at regulating their emotions and affect in response to changes in the environment (e.g., “is overly emotional”), and the negative emotionality subscale assesses children’s tendency to respond in an overly negative way to routine and novel environmental stimuli (e.g., “finds fault with everything”). These subscales were selected as the primary emotion regulation outcomes given their relative rigor according to an extensive review of all available emotion regulation measures (68). Psychometric support for the emotional self-control and negative emotionality subscales includes high internal consistency (α = 0.87–0.91) and test–retest reliability [*r* = 0.86–0.88; (62)]. The emotional self-control subscale contains 12 items, and the negative emotionality subscale contains 9 items for parent report and 8 items for teacher report. All items are rated on a four-point Likert-type scale (*never*, *sometimes*, *often*, *and almost always*). Higher raw scores indicate more difficulties with emotion regulation.

#### Emotion dysregulation inventory

An additional measure of emotion regulation was added to the study protocol in March 2018 given emerging data linking emotion regulation with the executive functions targeted by our protocols as described above. The Emotion Dysregulation Inventory (EDI) (69) assesses children’s emotion regulation based on parent report, and contains 13 items that are rated on a five-point Likert-type scale (e.g., “emotions go from 0 to 100 instantly”). Psychometric support for the EDI includes excellent internal consistency (α = 0.90–0.92), expected relations with other emotion regulation measures, and the ability to discriminate between children with known emotion regulation difficulties and their typically developing counterparts (70). Higher raw scores indicate more difficulties with emotion regulation.

Due to an administrative error, the parent *Emotion Regulation Checklist (ERC)* was administered instead of the EDI at pre-treatment for the first 62 participants. The EDI was administered at all other time points. The ERC (71) emotional lability subscale contains 15 items that are rated on a four-point Likert-type scale (e.g., “exhibits wide mood swings”). Psychometric support for the ERC includes high internal consistency (α = 0.98), discriminant validity relative to distinct constructs such as resilience, expected relations with other metrics of emotion regulation (*r* = 0.44–0.79), and the ability to differentiate between groups of children at-risk vs. not at-risk for emotional problems (71). Higher raw scores reflect more difficulties with emotion regulation. The ERC and EDI are strongly correlated [*r* = 0.53–0.64: (70)]; thus, we made the *a priori* decision to retain these participants and use their pre-treatment ERC data in the exploratory analyses. To equate the scaling across the ERC and EDI, we computed the proportion of the maximum possible score for each child for each measure at each time points.

### Intellectual functioning and socioeconomic status at pre-treatment

Pre-treatment IQ was estimated using the WISC-V Verbal Comprehension Index (72). Hollingshead (73) SES was estimated based on caregiver(s)’ education and occupation at pre-treatment.

### Informant expectancy questionnaires

#### Parent expectancies

Parent treatment-related expectancies were assessed via the NICT Expectations of Cognitive Training scale (74) at mid-treatment. The scale contains seven items that assess the extent to which parents expect cognitive training to improve their child’s functioning. Higher mean scores indicate higher expectancies (range = 1–7). The impact of parent expectancies on improvements in parent-reported emotion regulation during treatment was assessed via sensitivity analyses as described below.

#### Teacher expectancies

Teachers were not directly assessed for expectancies given our goal of obtaining ratings from teachers who were unaware that the children were receiving treatment. Instead, parents reported on the teachers’ knowledge of treatment participation on a study-created post-treatment blinding questionnaire. Based on parent report, all teachers remained masked to treatment allocation/group, whereas 37 of 94 (39.4%) teachers were told that the child was participating in an intervention [i.e., masked to treatment allocation but unmasked to study participation, creating the opportunity for expectancy effects; (66)]. The potential impact of teacher expectancies was assessed via sensitivity analyses as described below.

### Bayesian analyses

Traditional null hypothesis significance tests (*p*-values) were supplemented with Bayes Factors as recommended (75). Bayes Factors were added because they allow stronger conclusions by estimating the magnitude of support for both the alternative and null hypotheses (76). BF_10_ is the Bayes Factor (BF) indicating how much more likely the alternative hypothesis (H_1_) is relative to the null hypothesis (H_0_). Values >3.0 are considered moderate support for the alternative hypothesis (77). BF_01_ is the inverse of BF_10_ (i.e., BF_01_ = 1/BF_10_), and is reported when the evidence favors the null hypothesis (76). BF_01_ is interpreted identically to BF_10_ (>3 = moderate, >10 = strong, >100 = decisive evidence that ICT and CET produce equivalent changes in an outcome). We refer to findings of BF_10_ >3 as significant evidence *for* an effect (i.e., support for the alternative hypothesis of an effect at/above pre-specified evidentiary thresholds), and findings of BF_01_ >3 as significant evidence *against* an effect (i.e., support for the null hypothesis of no effect at/above pre-specified evidentiary thresholds). Both *p*-values and Bayes Factors are reported. We refer to effects as “marginally significant” when results indicate *p* < 0.05 but BF_10_ < 3.0 (i.e., when the effect is supported by null hypothesis testing but the Bayes Factor suggests evidentiary value below our prespecified threshold).

### Transparency and openness statement

Best practice guidelines for cognitive training studies were closely followed as detailed in Table 2. Trial outcomes and detailed data analytic plans for the CET vs. ICT randomized controlled trial were preregistered at https://osf.io/abwms. The analytic plan detailed in the preregistration was followed for the present study. All emotion regulation measures included in the study battery were analyzed and are reported here. Primary outcomes (effects on working memory, inhibitory control, and ADHD symptoms) and academic outcomes are reported in Kofler et al. (11) and Singh et al. (59), respectively, for subsets of the current sample. The deidentified raw data (.jasp) and results output (including analysis scripts and test statistics) are available for peer review as recommended (75): [https://osf.io/86fwu/]. We report how we determined our sample size, all data exclusions (if any), all manipulations, and all measures in the study.

### Data analysis overview

Data analyses were conducted with default priors using JASP version 0.14.1 (78). Our analytic plan included a 2 (between-subjects factor Treatment Group: CET vs. ICT) × 2 (within-subject factor Subscale: emotional self-control, negative emotionality) × 3 (within-subject factor Time: pre-, mid-, post-treatment) repeated measures ANOVA to examine treatment-related changes in emotion regulation based on parent report, with *post-hocs* following significant interactions and *a priori* planned contrasts to characterize the pattern of change over time separately for each treatment group. Similarly, teacher data were analyzed using a 2 (between-subjects factor Treatment Group: CET vs. ICT) × 2 (within-subject factor Subscale: emotional self-control, negative emotionality) × 2 (within-subject Time: pre-, post-treatment) repeated measures/mixed model ANOVA with the same *post hoc*/planned contrast plan.

## Results

### Power analysis

Power analysis using G*Power 3.1 (79) indicated that our sample size (*N* = 94), with α = 0.05, β = 0.80, and 3 time points (pre, mid, post), is powered to detect main effects of at least *d* = 0.29 and treatment group × time interaction effects of at least *d* = 0.34. Effects of these magnitudes were considered reasonable given evidence that (a) experimentally manipulating demands on working memory [*d* = 0.95; (10)] and inhibitory control [*d* = 0.52; (41)] both produce changes in emotion regulation that are at least moderate in magnitude; (b) CET produces large improvements in working memory [*d* = 0.96–1.20; (11, 34)]; and (c) ICT produces large improvements in stop-signal inhibitory control [*d* = 1.12; (11)]. Thus, the study is sufficiently powered to address its primary aims.

### Study retention, outliers, and missing data handling

Study retention was high for both CET (89% completers) and ICT (78%); completion rates did not differ based on treatment allocation (*p* = 0.33). The treatment groups also did not differ on missing data rates (*p* = 0.48–0.73); complete data were available for 80.5% of post parent, 70.1% of post teacher, and 66.2% of follow-up parent ratings. Missing data were determined to be missing completely at random (Little’s MCAR test: *p* > 0.99) and were imputed using expectation maximization based on all available data. This maximum likelihood-based approach has been shown to produce unbiased results for missingness rates at/above the current levels when data are missing at random (80), as was the case in the current study. Finally, all independent and dependent variables were screened for univariate outliers, defined as values greater than 3 *SD* outside the within-group mean. Outliers were corrected to the most extreme value within 3 *SD* of the mean; this process affected 2.3% of data points.

### Pre-treatment characteristics

Children randomized to ICT (*n* = 50) vs. CET (*n* = 44) did not differ from each other in parent- or teacher-rated emotion regulation or any of the pre-treatment characteristics shown in Table 1 (all BF_10_ ≤ 0.70, *p* > 0.05). Additionally, the treatment groups did not differ regarding comorbid diagnoses, training duration, or proportio nof children prescribed psychostimulants.

### Primary results

#### Tier 1: Parent-reported emotion regulation at immediate post-treatment

##### Behavior assessment scale for children-3 model

**FIGURE 3:**
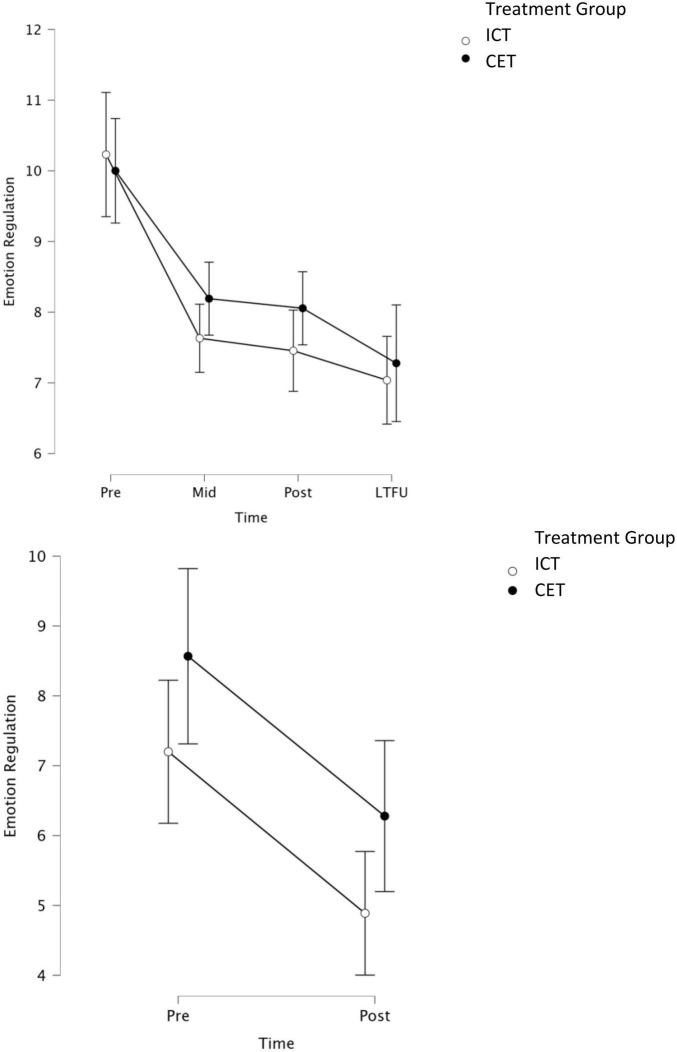
Graphical representation of parent-reported **(top)** and teacher-reported **(bottom)** models, with BASC-3 emotional self-control and negative emotionality subscales collapsed within each time point for each informant. Parent ratings were obtained at pre, mid, post, and LTFU. Teacher ratings were obtained at pre and 1–2 months post-treatment. LTFU, long-term follow-up (2–4 months after treatment concluded).

Consistent with our hypotheses, the 2 (between-subjects factor Treatment: CET, ICT) × 2 (within-subject factor Subscale: negative emotionality, emotional self-control) × 3 (within-subject factor Time: pre-, mid-, and post-treatment) repeated measures/mixed model ANOVA for parent-reported emotion regulation was significant for main effects of Time (BF_10_ = 8.04 × 10^13^, *p* < 0.001; η^2^_*p*_ = 0.28, *d* = 1.25) and Subscale (BF_10_ = 3.50 × 10^18^, *p* < 0.001; η^2^_*p*_ = 0.44, *d* = 1.77), and for the Time × Subscale interaction (BF_10_ = 3.00 × 10^35^, *p* = 0.002; η^2^_*p*_ = 0.06, *d* = 0.51; Figure 3). Contrary to our expectations, there was significant evidence *against* the Treatment × Time interaction (BF_01_ = 9.80, *p* = 0.32; η^2^_*p*_ = 0.01, *d* = 0.20), indicating that the CET and ICT groups showed equivalent reductions in parent-reported emotion regulation. Similarly, there was no significant main effect of Treatment or a Treatment × Subscale × Time interaction (all BF_10_ < 1, *p* ≥ 0.70). *A priori* planned contrasts indicated that both the CET and ICT groups demonstrated reductions in emotion dysregulation across both subscales from pre- to post-treatment (all BF_10_ ≥ 6.00, all *p* ≤ 0.002; *d*_*ICT*_ = 0.55 to 0.57; *d*_*CET*_ = 0.42 to 0.48). We also repeated the primary model using proportion of the total possible score for each subscale to account for the subscales containing different numbers of items. When using proportions, both the Subscale and Subscale × Time effects were no longer significant, suggesting that differences in subscales were due to scaling issues rather than differences in emotion regulation subcomponents. This interpretation is consistent with the planned contrasts, which indicated that both groups demonstrated improvement across both subscales from pre- to post-treatment.

##### Emotion dysregulation inventory model

Next, we examined the extent to which our primary results replicated using a narrowband measure of emotion regulation as described above. Results were consistent with the primary model, including a significant main effect of Time (BF_10_ = 3.95 × 10^41^, *p* < 0.001; η^2^_*p*_ 0.62, *d* = 1.28) and evidence against a Treatment × Time interaction (BF_01_ = 17.54, *p* = 0.70; η^2^_*p*_ = 0.004, *d* = 0.13).

#### Tier 2: Teacher-reported emotion regulation at 1–2 months post-treatment

Consistent with the parent-report model, the 2 (between-subjects factor Treatment: CET, ICT) × 2 (within-subject factor Subscale: negative emotionality, emotional self-control) × 2 (within subject factor Time: pre- and post-treatment) repeated measures/mixed model ANOVA was significant for the main effect of Time (BF_10_ = 1.22 × 10^6^, *p* < 0.001, η^2^_*p*_ = 0.19, *d* = 0.99). Similar to the parent-report model, there was evidence against a Treatment × Time interaction (BF_01_ = 5.08, *p* = 0.98, η^2^_*p*_ = 0.000006, *d* = 0.005), suggesting that children in both treatment conditions showed equivalent reductions in teacher-reported emotion regulation. There was a significant main effect of Subscale (BF_10_ = 4.35 × 10^17^, *p* < 0.001; η^2^_*p*_ = 0.61, *d* = 2.50) and a Time × Subscale interaction (BF_10_ = 5.03, *p* < 0.001; η^2^_*p*_ = 0.17, *d* = 0.91). However, in contrast to the parent model, both the Subscale and Time × Subscale effects remained significant when accounting for the different number of items per subscale using the proportion of total possible scores as described above, suggesting that patterns of improvement across the interventions were different for the distinct emotion regulation subcomponents. Indeed, *a priori* planned contrasts indicated that both treatment groups improved on the teacher-reported emotional self-control subscale from pre- to post-treatment (all BF_10_ > 10; all *p* ≤ 0.006; *d*_*ICT*_ = 0.68; *d*_*CET*_ = 0.35), whereas neither group exhibited significant changes in negative emotionality (BF_10_ ≤ 2; *p* ≥ 0.99).

#### Tier 3: Parent-reported emotion regulation at 2–4 months post-treatment

Additional analyses were conducted to probe for maintenance of effects based on parent report. These analyses involved repeating the parent pre/mid/post-treatment model above, this time adding follow-up as a fourth time point. Of primary interest were planned contrasts assessing (a) whether emotion dysregulation remained significantly below pre-treatment levels at follow-up (pre vs. follow-up), and (b) whether post-treatment gains were lost across the no-contact follow-up duration (post vs. follow-up). Reporting is truncated for readability. Results were consistent with the primary parent-report model reported above, including a main effect of Time (BF_10_ = 1.15 × 10^14^, *p* < 0.001; η^2^_*p*_ = 0.27, *d* = 1.22) and evidence against a Treatment × Time interaction (BF_01_ = 45.45, *p* = 0.52; η^2^_*p*_ = 0.008, *d* = 0.18). Pre-planned contrasts indicated that parent-rated emotion dysregulation across both subscales remained significantly reduced at follow-up relative to pre-treatment for both treatment groups (all BF_10_ > 10, all *p* ≤ 0.01; *d*_*ICT*_: emotional self-control = 0.66, negative emotionality = 0.60; *d*_*CET*_: emotional self-control = 0.67, negative emotionality = 0.50). Similarly, neither group demonstrated a significant loss in parent-rated emotional self-control or negative emotionality from post-treatment to follow-up, suggesting that the pre/post gains in emotion regulation were maintained at 2–4 months follow-up for both groups (all BF_10_ < 3, all *p* > 0.99).

#### Tier 4 sensitivity analyses: Expectancy effects, medication status, medication changes, maturation, and COVID protocol changes

Finally, we performed a series of exploratory analyses to examine the extent to which the significant reductions in emotion dysregulation during treatment may reflect an artifact of non-treatment processes (Table 3). The pattern, significance, magnitude, and interpretation of all results were unchanged when pre-treatment age, parent expectancies, teacher masking, medication status and changes, time between treatment completion and informants completing the post-treatment ratings, or COVID protocol status were added as covariates to the primary models. In all cases, the main effect of Time remained significant (all BF_10_ > 10, all *p* < 0.01; *d* = 0.50 to 1.15), suggesting that the improvements observed during treatment are unlikely to be due to these non-treatment factors. In no case did adding covariates result in a significant Treatment × Time interaction (all BF_01_ > 3; all *p* ≥ 0.52). Finally, none of the covariates showed significant main effects of Time or interacted with Treatment, Time, or Subscale, with one exception. In the teacher model, there was a significant main effect of age (BF_10_ = 2.96, *p* = 0.02; η^2^_*p*_ = 0.06) and an age × Subscale interaction (BF_10_ = 1.55 × 10^18^, *p* < 0.001; η^2^_*p*_ = 0.17), such that younger children exhibited greater improvements in teacher-reported emotional self-control (but not negative emotionality).

**TABLE 3:** Post-treatment outcome data and covariates for exploratory analyses.

Variable	ICT (*n* = 50)		CET (*n* = 44)		Cohen’s *d*	*BF*_01_	*p*
	*M*	*SD*	*M*	*SD*			
***Primary Outcomes***							
BASC-3 emotional self-control (total raw score)							
Parent	8.60	5.36	8.99	4.46	0.08	4.31	0.70, *ns*
Teacher	6.11	4.26	8.18	5.76	0.41	0.80	0.05, *ns*
BASC-3 negative emotionality (total raw score)							
Parent	6.31	3.48	7.12	3.27	0.24	2.57	0.25, *ns*
Teacher	3.67	3.25	4.38	4.08	0.19	3.13	0.35, *ns*
***Other Treatment-Related Variables***							
Medication changes (stop, no, add)	2, 33, 15		2, 29, 13		–	20.41	0.99, *ns*
Informant expectancies							
Parent NICT expectancy score (mean raw score)	4.53	1.01	4.67	0.74	0.15	3.65	0.47, *ns*
Teacher masked to study (yes/no)	30/20		27/17		–	4.02	0.89, *ns*
COVID-19 telehealth (yes/no)	11/39		10/34		–	4.69	0.93, *ns*
Time elapsed from treatment to post ratings (days)							
Parent	17.02	31.60	16.10	34.31	0.03	4.55	0.89, *ns*
Teacher	40.33	42.19	37.80	35.63	0.06	4.35	0.75, *ns*

CET, central executive training; ICT, inhibitory control training; BASC-3, behavior assessment scale for children; effect sizes and statistical tests for the BASC subscales reflect control for pre-treatment scores for the same subscale. BF, Bayes factor, BF_01_ is the odds ratio of the evidence favoring the null to the evidence favoring the alternative hypothesis. A value of 1 indicates that the data are equally likely under the null and alternative hypotheses, values >1 favor the null hypothesis that the groups are equivalent, and values >3 are considered statistically significant evidence of equivalence.

## Discussion

The current study was the first randomized controlled trial to compare the effects of training central executive working memory vs. inhibitory control on emotion dysregulation for children with ADHD. We hypothesized that both treatment groups would demonstrate reductions in emotion dysregulation during treatment, and that CET would be superior to ICT. Results indicated that both treatment groups exhibited moderate improvements in emotional control from pre- to post-treatment per both parent and teacher report, and both groups experienced moderate reductions in negative emotionality according to parent, but not teacher, report. Contrary to our expectations, reductions in emotion dysregulation from pre- to- post treatment were equivalent across the CET and ICT groups, as demonstrated by consistent evidence against a treatment × time interaction across all tested models. The use of CET and ICT as active, credible controls for one another is a significant improvement on previous trials that are unable to account for expectancy/placebo effects (66) in cognitive training interventions, most of which have proven unsuccessful in ameliorating ADHD symptoms and impairments in well-controlled clinical trials (49). However, the lack of significant treatment group × time interactions precludes us from confidently attributing improvements in emotion regulation to active treatment components of CET and ICT despite sensitivity analyses suggesting that the improvements during treatment were robust to all assessed threats to validity.

The significant reductions in parent- and teacher-reported emotion dysregulation that occurred during treatment were robust to control for most extraneous variables. Our findings were consistent with extant studies documenting improvements in emotion regulation following working memory training in neurotypical and clinical samples that did not include ADHD (42–46, 48). Additionally, the current study contributes to a small, mixed literature, in which some studies find that training inhibitory control improves emotion regulation for neurotypical children and adults with emotion regulation difficulties (41, 48) but not for neurotypical adults (47). There is little research on emotion regulation outcomes when training executive functions in children with ADHD, but our findings conflict with Tamm et al. (50) and Tamm et al. (51) who found no impact of a play-based metacognitive attention training on emotion regulation. However, this discrepancy is unsurprising given the difference in training targets relative to the current study. That is, directly training a specific executive function provides a more potent treatment dose than attempting to target executive functioning more broadly (49), and CET and ICT target specific cognitive functions that are linked with ADHD-related emotion dysregulation (7–10). Thus, it appears possible that both working memory and inhibitory control may be causally linked with emotion regulation difficulties in ADHD. At the same time, clinical implications should be considered tentative because we did not include an untreated control group to conclusively rule out spontaneous recovery as an alternative explanation for the significant improvements associated with both active treatments.

Although the current study cannot conclusively rule out maturation/spontaneous recovery as an explanation for the significant improvements in emotion regulation that occurred during treatment for both groups, this explanation appears unlikely based on extant literature and when contextualized with other results from the RCT. For example, children often experience *more* difficulties with emotion regulation between the ages of 9 and 13 than they do in early childhood (81–83). In other words, maturation effects, if present, might be expected to produce *increases* in emotion regulation difficulties rather than the decreases observed in the current study. Thus, it seems unlikely that we would expect to see spontaneous improvements in emotion regulation over this time period in the absence of targeted intervention. Notably, however, to our knowledge, no study to date has reported on the spontaneous development of children’s emotion regulation over the relatively short duration covered by our active treatment phase (i.e., 10 weeks).

Similarly, if the observed improvements in emotion regulation were artifacts of maturation, spontaneous recovery, or other factors unrelated to the tested interventions, it seems unlikely that we would have obtained the specific patterns of improvement that were observed across informants and time points. In particular, it seems reasonable to conclude that the pattern of significant pre/post improvement followed by the non-significant post/follow-up change makes these alternative explanations unlikely. Stated differently, it would seem to be a logical stretch to argue that children experienced an acute episode of maturation/spontaneous recovery that, coincidentally, temporally coincided with the active treatment component and then, coincidentally, abruptly stopped when treatment was completed. Similarly, the maintenance of gains at follow-up, combined with our sensitivity analyses, also appears to effectively rule out expectancy effects as an alternative explanation for the observed improvements. That is, our understanding is that expectancy effects are time limited rather than producing lasting change (66), in which case an expectancy hypothesis would not be able to account for the lasting improvements observed in the current study.

Alternatively, it is possible that the improvements observed during treatment were attributable to non-specific or shared components of the treatments rather than the treatment targets specifically. For example, participants in both conditions participated in comprehensive psychoeducational evaluations, which included feedback sessions that provided psychoeducation about ADHD and associated difficulties as needed. However, psychoeducation alone has failed to improve ADHD-related emotion dysregulation and higher doses of psychoeducation may be iatrogenic for ADHD-related treatment outcomes (84–86). Additionally, both CET and ICT included routine contact with the study team. Extant literature documents the non-specific benefits of supportive clinician contact (87), which has also demonstrated incremental value in trials of internet-based psychosocial interventions (88, 89). However, it is unlikely that improvements are solely attributable to clinician contact given that significant treatment × time interaction effects have been found for most other studied outcomes from this trial, including ADHD symptoms, academic outcomes, and organizational skills (11, 59, 90). In essence, there does not appear to be a compelling argument that non-specific clinician contact would specifically impact some but not most studied outcomes—especially given that teachers also reported improvements despite no contact from the study team.

Beyond the potential but tentative clinical intervention implications, results of the current study add to research documenting cross-sectional links between inhibitory control and ADHD-related emotion regulation (9, 40), and extend these findings by suggesting that these relations may be causal. However, the equivalent reduction in emotion dysregulation for both treatment groups is somewhat inconsistent with cross-sectional evidence that working memory but not inhibition uniquely predicts ADHD-related emotion dysregulation when included in the same model (8). Future work specifically examining the extent to which (a) improvements in working memory or inhibitory control covary with improvements in emotion regulation; and (b) these performance improvements are reflected at the cortical level will be important for furthering our understanding of the role of these executive functions in children’s emotion regulation skills.

### Limitations

The present study demonstrates several strengths, including a carefully characterized sample of children with ADHD with and without comorbidities, outcome ratings from multiple informants masked to treatment allocation, and intervention groups that served as active, credible controls for one another (66). However, some limitations warrant consideration when interpreting results. First, ICT was developed as an ideal active, credible control for CET in consideration of expected effects on the trial’s primary clinical outcomes (ADHD symptoms) given experimental evidence implicating working memory (37, 91), but not inhibitory control (92), as a causal mechanism underlying core ADHD symptoms. However, given experimental evidence for functional, if not causal, roles for both working memory and inhibitory control on emotion regulation (10, 41), it will be necessary for future trials to include a third treatment arm that targets processes(es) unrelated to children’s emotion regulation skills. Given that previous studies suggest that executive functions, particularly working memory, exert direct effects on emotion regulation as well as indirect effects via ADHD inattentive and hyperactive/impulsive symptoms, future trials should consider the extent to which improvements in emotion regulation represent a direct outcome of executive function training vs. a downstream outcome of improved ADHD symptoms.

Additionally, the clinical diversity of the sample was useful given that comorbidity is the rule rather than the exception in individuals with ADHD [e.g., (93)], but the inclusion of comorbidities may limit the specificity of these findings regarding children with only ADHD. Unexpectedly, children with ADHD whose families self-selected out of the treatment phase had moderately higher emotion regulation difficulties based on teacher but not parent report. Despite finding medium to large improvements in emotion regulation for treated children, it is possible that larger effects would have been detected if more severely dysregulated children were retained in the trial and/or if we recruited specifically for children with emotion regulation difficulties. Finally, most participants in the current trial identified as White/non-Hispanic. Future studies should recruit samples with larger proportions of historically excluded racial/ethnic groups to ensure that results generalize to these groups.

### Clinical and research implications

Taken together, results of this double-blind randomized controlled trial were consistent in documenting significant improvements in emotion regulation for children with ADHD that persist at least 2–4 months after treatment termination and are not likely artifacts of any assessed threats to validity. If results of the current study are consistent with future studies including an additional control treatment that targets a mechanism that would not be expected to affect emotion regulation, it would appear likely that working memory and inhibitory control are potentially functionally linked with emotion regulation difficulties in ADHD, consistent with prior experimental evidence in ADHD and non-ADHD samples (10, 41). At the same time, implications for clinical practice should be considered tentative because we did not include an additional control treatment that targets a mechanism that would not be expected to affect emotion regulation. The incremental value of adding CET and/or ICT to extant evidence-based treatments for emotion dysregulation should be examined in future work, as the combination of improving the underlying neurocognitive foundation and directly training emotion regulation skills may prove more beneficial for improving functioning for children with ADHD than treating emotion dysregulation in isolation (54, 55).

## Data availability statement

The datasets presented in this study can be found in online repositories. The names of the repository/repositories and accession number(s) can be found below: Open Science Framework (OSF) https://osf.io/86fwu/.

## Ethics statement

The studies involving human participants were reviewed and approved by the Florida State University Human Subjects Committee. Written informed consent to participate in this study was provided by the participants’ legal guardian/next of kin. Written informed consent was obtained from the minor(s)’ legal guardian/next of kin for the publication of any potentially identifiable images or data included in this article.

## Author contributions

NG was the lead investigator for analysis and interpretation of the data and drafting of the work. MK was the principal investigator for funding acquisition and had a supporting role in revision of the manuscript. All authors made substantial contributions to the conception of the work, revision of the manuscript, and the acquisition of data for the work, and provided approval for publication of the content.
